# Self-Assembly of Three-Dimensional Hyperbranched Magnetic Composites and Application in High-Turbidity Water Treatment

**DOI:** 10.3390/molecules29153639

**Published:** 2024-08-01

**Authors:** Yuan Zhao, Qianlong Fan, Yinhua Liu, Junhui Liu, Mengcheng Zhu, Xuan Wang, Ling Shen

**Affiliations:** 1 School of Chemistry & Chemical Engineering, College of Basic Medicine and Forensic Medicine, Henan University of Science and Technology, Luoyang 471000, China; 2 School of Life Science, Jiangsu Normal University, Xuzhou 221116, China

**Keywords:** self-assembly, hyperbranched polymer, Fe_3_O_4_, magnetic flocculation, EDLVO

## Abstract

In order to improve dispersibility, polymerization characteristics, chemical stability, and magnetic flocculation performance, magnetic Fe_3_O_4_ is often assembled with multifarious polymers to realize a functionalization process. Herein, a typical three-dimensional configuration of hyperbranched amino acid polymer (HAAP) was employed to assemble it with Fe_3_O_4_, in which we obtained three-dimensional hyperbranched magnetic amino acid composites (Fe_3_O_4_@HAAP). The characterization of the Fe_3_O_4_@HAAP composites was analyzed, for instance, their size, morphology, structure, configuration, chemical composition, charged characteristics, and magnetic properties. The magnetic flocculation of kaolin suspensions was conducted under different Fe_3_O_4_@HAAP dosages, pHs, and kaolin concentrations. The embedded assembly of HAAP with Fe_3_O_4_ was constructed by the N–O bond according to an X-ray photoelectron energy spectrum (XPS) analysis. The characteristic peaks of –OH (3420 cm^−1^), C=O (1728 cm^−1^), Fe–O (563 cm^−1^), and N–H (1622 cm^−1^) were observed in the Fourier transform infrared spectrometer (FTIR) spectra of Fe_3_O_4_@HAAP successfully. In a field emission scanning electron microscope (FE-SEM) observation, Fe_3_O_4_@HAAP exhibited a lotus-leaf-like morphological structure. A vibrating sample magnetometer (VSM) showed that Fe_3_O_4_@HAAP had a relatively low magnetization (*Ms*) and magnetic induction (*Mr*); nevertheless, the ferromagnetic Fe_3_O_4_@HAAP could also quickly respond to an external magnetic field. The isoelectric point of Fe_3_O_4_@HAAP was at 8.5. Fe_3_O_4_@HAAP could not only achieve a 98.5% removal efficiency of kaolin suspensions, but could also overcome the obstacles induced by high-concentration suspensions (4500 NTU), high pHs, and low fields. The results showed that the magnetic flocculation of kaolin with Fe_3_O_4_@HAAP was a rapid process with a 91.96% removal efficiency at 0.25 h. In an interaction energy analysis, both the U*_DLVO_* and U*_EDLVO_* showed electrostatic repulsion between the kaolin particles in the condition of a flocculation distance of <30 nm, and this changed to electrostatic attraction when the separation distance was >30 nm. As Fe_3_O_4_@ HAAP was employed, kaolin particles could cross the energy barrier more easily; thus, the fine flocs and particles were destabilized and aggregated further. Rapid magnetic separation was realized under the action of an external magnetic field.

## 1. Introduction

In general, the turbidity of natural rivers and lakes increases as rainfall continuously scours their surfaces, which also greatly increases the risk of unsafe drinking water [1]. In addition, high turbidity also appears in the treatment process of waterworks [2], and agriculture and industry produce wastewater from paint [3], microplastic [4], dye [5], papermaking [6] beneficiation, and livestock and poultry breeding [7]. The colloidal particles in high-turbidity water usually exhibit the characteristics of electric charge, a small particle size, and a large specific surface area [8]; also, the particles carry harmful pollutants such as heavy metals, pathogens, and toxic organics [9,10]. Therefore, the treatment of high-turbidity water is essential and has enormous challenges.

For the treatment of high-turbidity water, the common methods mainly focus on physical, chemical, and biological methods. Physical methods mainly include centrifugation, filtration, gravity sedimentation, etc., which are usually time-consuming and energy-demanding; thus, they are difficult to use for large-scale applications.

Biological methods mainly select some filter-feeding fish or plankton, but their growth properties are difficult to control, as are the application effects [11]. In chemical methods, flocculation can be induced by chemicals, both inorganic and organic, or by microorganisms. The flocculation process can occur in response to environmental stress, certain pHs, nitrogen, and dissolved oxygen. In this process, the shape, size, and composition of flocs can be very dependent on the colloidal particles and flocculant [12,13]. Thus, selecting the appropriate and ideal flocculant is the key to obtaining a high flocculation efficiency in the treatment process.

Magnetic flocculation has emerged as a new technology, in which the separation process is achieved via the intrinsic paramagnetic movement of magnetic particles tagged with target colloidal particles. The magnetic flocculation process has the advantages of being convenient, maneuverable, environmentally friendly, quickly achieving separation, reducing or eliminating the leaching of pollutants, and facilitating its reuse [14,15,16]. During the application of magnetic flocculation, magnetic composites can react with the target pollutants through charge neutralization, adsorption bridge, double electric layer compression, and sweeping, so as to generate a high magnetic concentration and high-density composite flocs, which can achieve rapid settlement through the influence of gravity or an external magnetic field [17]. The core technology of magnetic flocculation mainly depends on the magnetic particles that are employed. Among magnetic particles, the common magnetic carriers are the magnetic particles of Fe_3_O_4_, which have the advantages of being available, inexpensive, and having a high magnetic responsiveness [18,19,20]. The flocculating materials for magnetic assembly are concentrated in selection from conventional flocculants. Recently, stereo-structured polymers have attracted wide attention because of their ability to overcome the structural instability of conventional linear or chain polymers, and hyperbranched polymers (HBPs) are typical examples among them.

HBPs applied as a new type of highly branched polymer have quickly attracted much attention, owing to their highly three-dimensional (3D) and highly branched topologies, high branches, and high density of surface functional groups [21,22,23]. Compared with normal linear polymers (LPs), the main characteristics of HBPs include a good solubility and low viscosity; special 3D topological structures that can prevent wounds and aggregation, resulting in some completely different properties from LPs; and numerous functional groups along the periphery of HBPs, with the properties of a higher solubility and easy modification/adjustment by changing the groups along the periphery [24]. The functional groups mainly include amine (–NH_2_), hydroxyl (–OH), and carboxyl (–COOH) groups, which increase the adsorption sites of HBPs [25]. Moreover, the branched chains and terminal functional groups of HBPs’ components are adjustable, which broadens their application range [26]. Up to now, there are several types of HBPs or modified substances, including polyamide amine (PAMAM) [27], hyperbranched pyridylphenylene polymer (PPP) [28], hyperbranched polyglycidol (HbPGL) [29], and hyper-branched polyethylenimines (HPEIs) [30], which are usually employed for assembling and fabricating in water treatment.

Magnetic composites assembled from HBPs and magnetic materials improve the stability and reusable performance of HBPs, successfully making up for their shortcomings. Within these composites, magnetic Fe_3_O_4_ is evenly dispersed, and then the outer molecules are iteratively grafted to form a hyperbranched polymer, which ensures their magnetic response ability [31,32]. Meanwhile, the magnetic core and polymer are tightly bound together via a grafting reaction, and the hyperbranched carbon chains and terminal functional groups show a great ability in solid–liquid separation under the action of an external magnetic field [31]. It is known that flocculants with a high molecular weight and rich active groups are more effective in exhibiting the function of adsorption bridging ability [33,34]. Fortunately, magnetic hyperbranched polymers acquire the characteristics of both magnetic particles and dendritic macromolecular polymers.

In this research, a new type of three-dimensional hyperbranched magnetic composite (Fe_3_O_4_@HAAP) was obtained using a self-assembly strategy of hyperbranched amino acid polymer (HAAP) and naked Fe_3_O_4_ particles. Thus the magnetic flocculation properties and mechanisms were investigated. In order to explore the characteristics of Fe_3_O_4_@HAAP, the morphology, structure, configuration, chemical composition, charged characteristic, and magnetic and thermal properties were explored. The magnetic flocculation performance of Fe_3_O_4_@HAAP was evaluated in high-turbidity wastewater, taking into account factors like the dosage, pH, and kaolin concentration. In addition, the Derjaguin–Landau–Verwey–Overbeek (DLVO theory) was used to describe the interaction force between charged aggregates in order to discuss the reaction mechanism of the material.

## 2. Result and Discussion

### 2.1. Characterization of Fe_3_O_4_@HAAP

#### 2.1.1. FTIR and XPS Spectra

**Figure 1:**
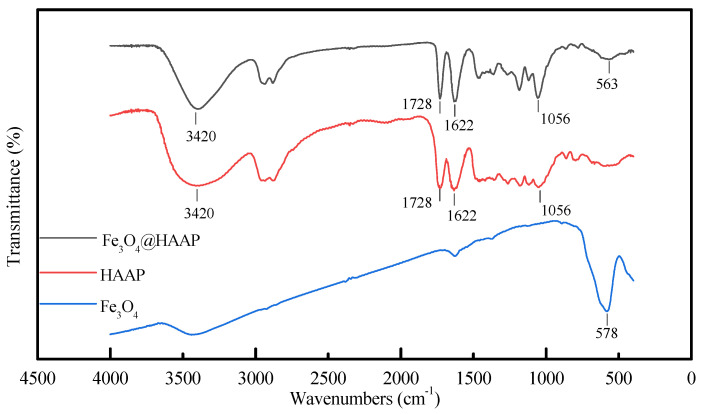
The FTIR spectra of Fe_3_O_4_, HAAP, Fe_3_O_4_@HAAP.

The FTIR spectra of Fe_3_O_4_@HAAP, HAAP, and Fe_3_O_4_ are shown in Figure 1. An obvious Fe-O stretching vibration peak was observed at 578 cm^−1^. At 563 cm^−1^ of Fe_3_O_4_@HAAP, a comparable, slightly smaller peak can be seen. This verified that the HAAP had been successfully coated on the surface of Fe_3_O_4_ [35]. The peaks at 3420 cm^−1^, 1728 cm^−1^, and 1622 cm^−1^ are attributable to the stretching vibration of the –OH group, C=O band, and N–H band [36,37,38]. The weakening of these peaks also demonstrated the success of Fe_3_O_4_@HAAP assembly.

**Figure 2:**
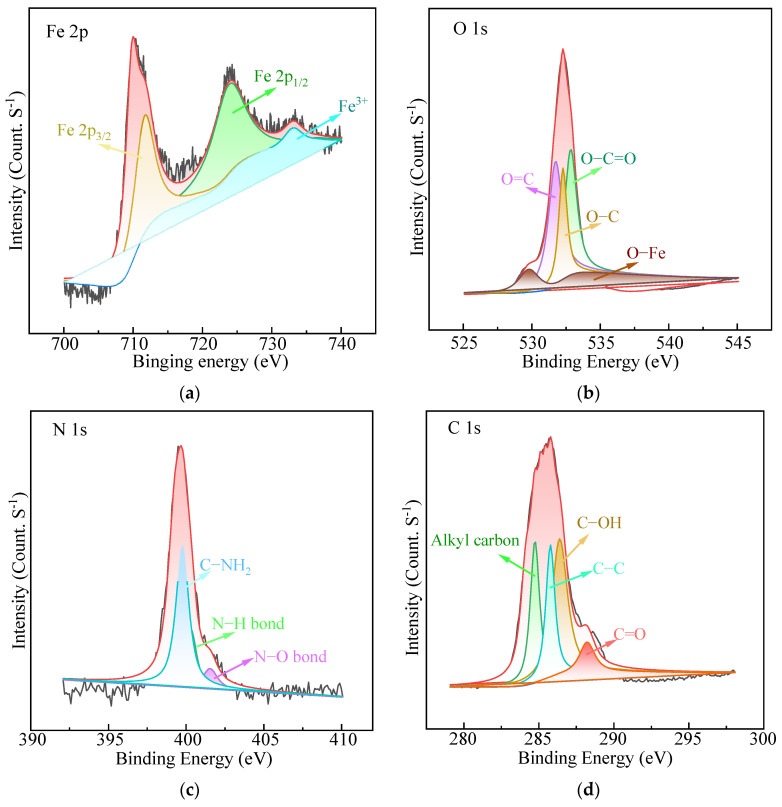
The XPS spectra of Fe_3_O_4_@HAAP: (**a**) Fe 2p spectrum, (**b**) O 1s spectrum, (**c**) C 1s spectrum, (**d**) N 1s spectrum.

The XPS spectra of Fe_3_O_4_@HAAP are presented in Figure 2. As to the Fe 2p high-resolution spectrum (Figure 2a), the two typical peaks at 724.6 and 710.8 eV correspond to Fe 2p_1/2_ and Fe 2p_3/2_. Specifically, the two peaks of Fe 2p_3/2_ and Fe 2p_1/2_ were deconvoluted into Fe^3+^ and Fe^2+^ ions. The small satellite peak at 733.3 eV could be assigned to Fe^3+^ [39]. Figure 2b shows the O 1s high-resolution spectra, with the four peaks at 532.9, 531.8, 532.3, and 529.8 eV attributed to O–C=O, O=C, O–C, and O–Fe, respectively [40,41,42]. In addition, three prominent peaks were observed at 286.4, 285.8, and 288.2 eV, corresponding to C–OH, C–C, and C=O (Figure 2c) [43,44]. Three peaks were identified in the N 1s spectrum (Figure 2d) at binding energies of 400.3, 401.5, and 399.8 eV, which belonged to N–H, N–O, and C–NH_2_ [21,42,45].

#### 2.1.2. SEM and EDS

**Figure 3:**
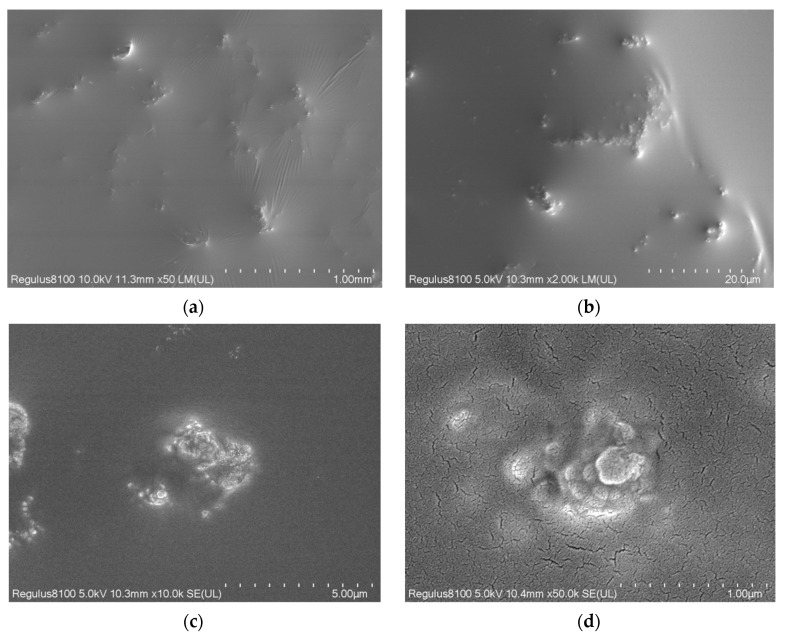
SEM images of Fe_3_O_4_@HAAP: (**a**) ×50; (**b**) ×2000; (**c**) ×10,000; (**d**) ×50,000.

The morphology of Fe_3_O_4_@HAAP was examined by SEM, and the images at various magnifications are shown in Figure 3. The surface of Fe_3_O_4_@HAAP was covered by an oil-like film, and there were scattered patches (Figure 3a–c). This was due to the presence of the organic coating after the assembly of Fe_3_O_4_ and HAAP. In the ×50,000 observation, Fe_3_O_4_@HAAP exhibited a lotus leaf-like morphological structure (Figure 3d). Two points were randomly selected on the surface of the Fe_3_O_4_@HAAP for energy spectrum analysis, and the specific results can be observed in Table 1. Except for C and O elements, Fe element content was dominated by the others (16%). In addition, there was a small amount of Si element (3.2%), which came from the raw materials (tetraethylxysilane) in the Fe_3_O_4_@HAAP assembly process.

#### 2.1.3. VSM and Zeta Potential

**Figure 4:**
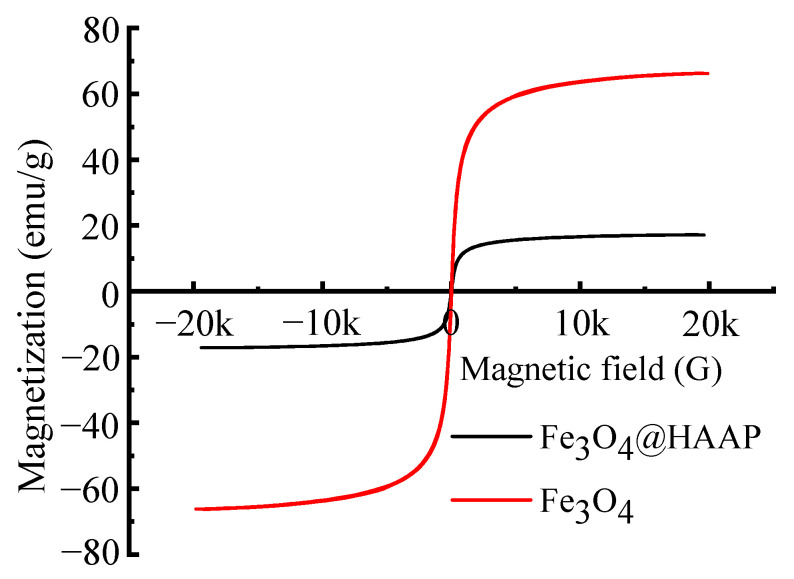
The magnetization hysteresis loops of Fe_3_O_4_ and Fe_3_O_4_@HAAP.

The hysteresis loop is a curve formed by the magnetic induction intensity of the sample with the change in the magnetic field, which represents the magnetic properties of the material. Figure 4 shows the magnetization hysteresis loops of Fe_3_O_4_ and Fe_3_O_4_@HAAP under varying magnetic field intensities. The saturation magnetization (*Ms*) of Fe_3_O_4_ and Fe_3_O_4_@HAAP was 66.13 emu/g and 17.1 emu/g, respectively. The reason for this phenomenon was that during the assembly of Fe_3_O_4_@HAAP, a non-magnetic shell was formed on the surface. In addition, the coercive force (*Hc*) and remanent magnetic induction (*Mr*) of Fe_3_O_4_ and Fe_3_O_4_@HAAP were 13.6 Oe, 1.35 emu/g and 17.49 Oe, 0.57 emu/g, respectively. The high value of *Hc* but low *Mr* showed that Fe_3_O_4_@HAAP exhibited superparamagnetic properties and was easily affected by magnetic fields, resulting in quick magnetic separation in application.

**Figure 5:**
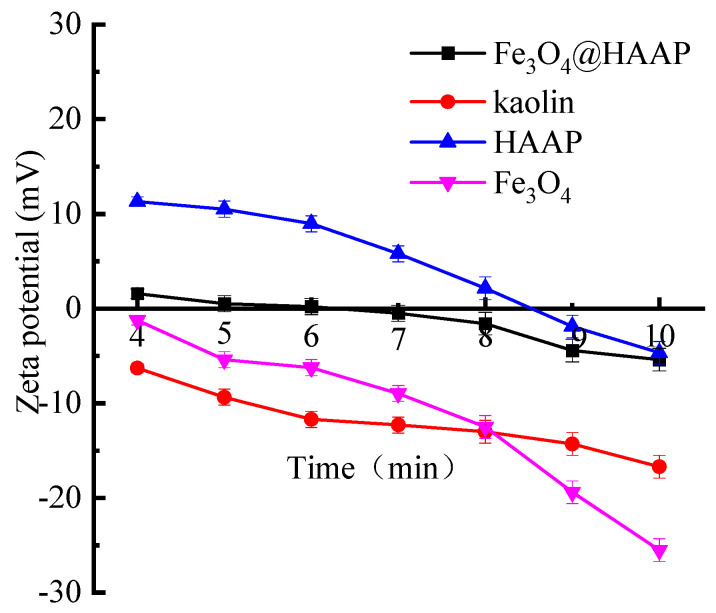
Zeta potential of kaolin solution, Fe_3_O_4_, HAAP, and Fe_3_O_4_@HAAP.

The zeta potentials of kaolin solution, Fe_3_O_4_, HAAP, and Fe_3_O_4_@HAAP are displayed in Figure 5. The zeta potential of kaolin was negative and the absolute value increased with the increase in pH. Due to the crystal structure formed by the alternately arranged Si–O tetrahedral layer and Al–O octahedral layer and the active group type on the surface being O^2−^, the surface of kaolin has a negative charge. For Fe_3_O_4_@HAAP, the zeta potential was −5.39~1.58 mV and the isoelectric point was about 6. These values were all between Fe_3_O_4_ and HAAP, which, because of the successful assembly of two materials, resulted in a change in the zeta potential of Fe_3_O_4_@HAAP.

### 2.2. Application in Wastewater Treatment

#### 2.2.1. Simulated Wastewater

**Figure 6:**
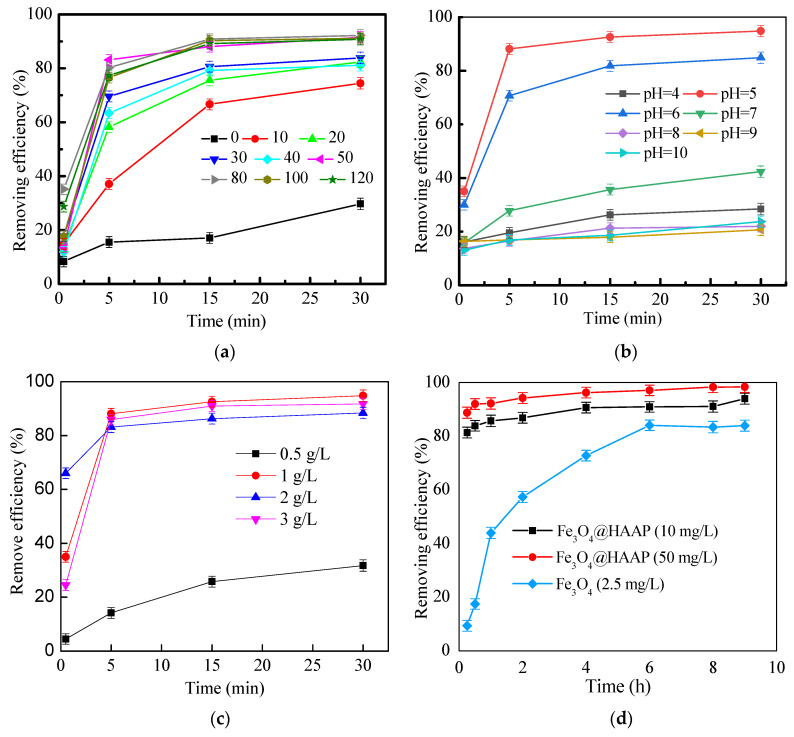
Removing efficiency of Fe_3_O_4_@HAAP on kaolin solution under different conditions: (**a**) Fe_3_O_4_@HAAP dosage, (**b**) pH, (**c**) kaolin concentration, (**d**) reaction time.

Fe_3_O_4_@HAAP was applied to treat kaolin simulated wastewater, and the different conditions were explored. The experimental results are shown in Figure 6. In order to compare the effects of different dosages, 0~120 mg/L of Fe_3_O_4_@HAAP was added to the wastewater, respectively. When the dosages were below 40 mg/L, the removing efficiency was less than 85% at 30 min. While the dosages further exceeded 50 mg/L, the removing efficiency was over 90% at 30 min. The removing efficiency reached 91.53% when the dosage was 50 mg/L (Figure 6a). Under acidic conditions, the zeta potential of Fe_3_O_4_@HAAP was positive and the surface was positively charged. The material could be combined with the negatively charged kaolin by electroneutralization to achieve the agglomeration. However, the low-dose flocculant was not enough to realize the electric neutralization mechanism, and reduced the possibility of collision, which caused the poor flocculation effect. In addition, the excessive dosage caused the contact site between the kaolin particles and the flocculant to be covered, the bridging effect was weakened, and the electrostatic repulsion between the floc was dominant, which made the floc unstable and was not conducive to the agglomeration of particles.

Figure 6b shows the effect of different pHs on the removing efficiency. When pH was 5, the removing efficiency was optimal and the value was 88.11% at 5 min, while it reached 94.83% at 30 min. And there was a significant difference (*p* < 0.05) in the removing rate between the two periods, which indicates that the flocculation process is not in equilibrium at 5 min. When Fe_3_O_4_@HAAP was applied in the strong acid condition, H^+^ in the solution inhibited the dissociation of carboxyl groups, thus reducing the complex ability of the material and weakening the flocculation effect. However, –COO^−^ ions could be ionized from the –COOH and become negatively charged under alkaline conditions. This caused the ionization of the carboxyl group on the surface of Fe_3_O_4_@HAAP, which resulted in its zeta potential becoming negative and the absolute value gradually increasing, thus increasing the electrostatic repulsion between Fe_3_O_4_@HAA and kaolin particles, and reducing the flocculation effect.

As shown in Figure 6c, the removing efficiency exhibited significant differences when Fe_3_O_4_@HAAP was used to treat kaolin suspension. When the kaolin concentration was 1.0 mg/L, Fe_3_O_4_@HAAP showed the best removal properties and the value was 94.83%. However, when the kaolin concentration was 0.5 mg/L, the removing efficiency was only 31.74%. The excessive concentration of kaolin also resulted in a slight decrease in the removing efficiency. The reason was that the amount of flocculant was higher than the pollutant, and then the spatial resistance of the polymer and the electrostatic repulsion between the floc caused the stability of the formed floc to deteriorate. The particle aggregation ability was also reduced, and the degree of fragmentation was increased, which resulted in a poor processing effect. However, excessive kaolin also led to a decline in treatment performance because of the insufficient binding site of the flocculant, resulting in the positive charge of Fe_3_O_4_@HAAP, which could not completely neutralize the negative charge of kaolin.

Different dosages of Fe_3_O_4_@HAAP were added to treat kaolin, and the results can be observed in Figure 6d. Fe_3_O_4_ was added for the control group. The results showed that the flocculation of kaolin with Fe_3_O_4_@HAAP was a rapid process, and that the removing efficiencies were 83.85% and 91.96% at 0.25 h when the dosages of Fe_3_O_4_@HAAP were 10 mg/L and 50 mg/L, respectively. After analysis, the flocculation kinetics conformed to the Smoluchowski classical model. In the experiment, the small kaolin particles first rapidly aggregate and then flocculate through bridging into larger flocs. But for Fe_3_O_4_@HAAP, the low dosage weakened the interaction between the flocculant and the kaolin. This was not only insufficient to produce a full collision between flocculant molecules and kaolin particles, but also insufficient to realize an electric neutralization mechanism. Moreover, appropriate addition made the positive charge of the magnetic material and the negative charge of kaolin electric neutralization particle collision sufficient, and combined with the adsorption bridge to achieve rapid flocculation [46]. However, with the excessive dosage, both the spatial resistance of the polymer and electrostatic repulsion between the flocs led to instability, reduced the particle aggregation degree, and increased the fragmentation degree, thus affecting the flocculation kinetics and flocculation performance [47].

#### 2.2.2. Actual Water

**Figure 7:**
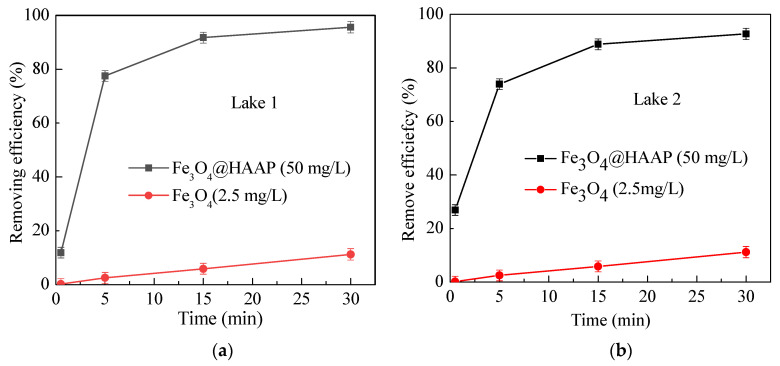
Removing efficiency of Fe_3_O_4_@HAAP on actual water: (**a**) Lake 1, (**b**) Lake 2. The Fe_3_O_4_@HAAP dosage was 50 mg/L, pH = 5.

In addition to the simulated wastewater samples, actual water at its natural pH and temperature conditions was used in determining the effects of Fe_3_O_4_@HAAP. An addition of Fe_3_O_4_ was used as a comparison. Different from the simulated water samples, there are a variety of complex substances, such as plankton, chemical components existing in nature, which may influence the results of experiments. Samples from two lakes were collected to verify the practicability of Fe_3_O_4_@HAAP, and the results are shown in Figure 7. Fe_3_O_4_@HAAP showed a significant removing effect in both lake samples (Figure 7a,b). The removing efficiencies were 95.6% and 92.7% at 30 min, respectively. The results indicated that Fe_3_O_4_@HAAP could overcome the influence of interfering substances in natural water and maintain an excellent magnetic flocculation effect.

#### 2.2.3. Recycling and Reuse

**Figure 8:**
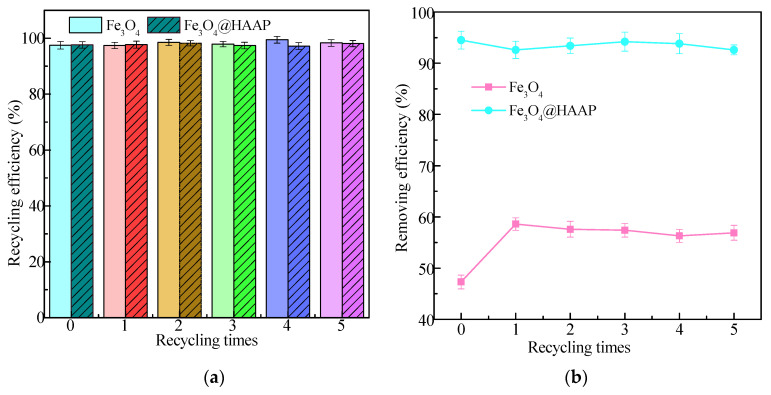
The recycling efficiency (**a**) and removing efficiency (**b**) of Fe_3_O_4_ and Fe_3_O_4_@HAAP on kaolin treatment under 5 recycling times, the colors correspond to different recycling times.

It is well known that the recyclability and reusability of materials are key factors in reducing costs. Fe_3_O_4_ and Fe_3_O_4_@HAAP were separated from solution by a magnet and the reuse performance was explored via treating kaolin solution. As shown in Figure 8a, the recycling efficiency of both Fe_3_O_4_ and Fe_3_O_4_@HAAP remains above 97% and there was no significant difference (*p* > 0.05) in the efficiency of each recycling efficiency with only a small amount of material loss, which may be due to incomplete material separation during the flocculation–separation process. In the kaolin treatment process, although Fe_3_O_4_@HAAP was repeatedly applied five times, the removing efficiency decreased slightly each time, with no significant difference (*p* > 0.05). Therefore, Fe_3_O_4_@HAAP maintained high removing efficiency even after five cycles, with a value of 92.6% (Figure 8b). The results showed that Fe_3_O_4_@HAAP had excellent stability, could be reused multiple times, and maintained excellent treatment effects, which made the material a potential candidate for the treatment of high-turbidity wastewater.

### 2.3. Interaction Energy Analysis

In the DLVO theory calculation model, the van der Waals force is the dipole between the molecules or the atoms of two particles, resulting in an interaction between the atoms or molecules of the two particles. Electrostatic force refers to the force generated by the interaction of double electric layers between particles. In DLVO theory, the total interaction energy between particles depends on the interaction potential energy generated by the van der Waals interaction (U*_VDW_*) and the electrostatic interaction force energy caused by the compression double layer (U*_EI_*) [48]. However, the extended DLVO (EDLVO) theory revises the classical DLVO theory by supplementing the Lewis acid–base polar (AB) force. There is a polar force between particles that can explain the repulsive force due to hydration and the electrostatic force at the hydrophilic and hydrophobic interfaces [49]. Therefore, in EDLVO theory, the total interaction energy between particles is determined by U*_VDW_*, U*_EI_,* and U*_AB_* [50].

**Figure 9:**
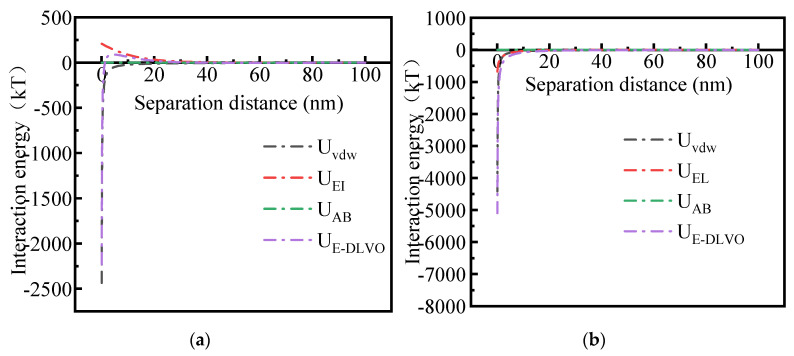
The interaction energy between Fe_3_O_4_@HAAP and kaolin: (**a**) kaolin–kaolin; (**b**) Fe_3_O_4_@HAAP–kaolin. pH = 5.

The interaction potential energy between Fe_3_O_4_@HAAP and kaolin was analyzed via DLVO and EDLVO theory, and the results are shown in Figure 9. As observed in Figure 9a, for the two interacting kaolin particles, when the separation distance was less than 30 nm, the value of U*_DLVO_* between kaolin was positive, which indicated that the force between particles behaved as an electrostatic repulsion. However, when the separation distance was greater than 30 nm, the value turned negative, which meant the force between particles gradually changed to electrostatic attraction [51]. In addition, when the separation distance is in the range of 0–10 nm, there is a repulsive barrier between the interaction particles. The presence of the barrier meant that the repulsion potential of kaolin in the solution was greater than the kinetic energy of Braun movement, and kaolin in the solution was suspended, without instability or aggregation. Otherwise, the value of U*_EDLVO_* between particles was similar to that of U*_DLVO_*; this was because, in the kaolin solution, the mutual potential energy between the particles is still contributed by U*_EI_*.

Figure 9b shows the interaction potential energy of Fe_3_O_4_@HAAP–kaolin, represented by the dissociation curve, when Fe_3_O_4_@HAAP was added to the kaolin solution. The value of U_DLVO_ between Fe_3_O_4_@HAAP and kaolin was negative, due to the same negative values of U*_vdw_* and U*_EI_*, and the electrostatic force was manifested as an attractive force [52]. Hence, with the addition of Fe_3_O_4_@HAAP, the external magnetic field would cause the energy barrier of kaolin particles to be crossed, and the particles would be destabilized and aggregated. Using more accurate EDLVO theory, it is found that the value of U*_EDLVO_* in the system was also negative throughout the separation distance, and there was still no energy barrier. Based on the results, it was indicated that the influence of Lewis acid–base interaction energy U*_AB_* on the total interaction energy was negligible.

## 3. Materials and Methods

### 3.1. Materials

All chemicals used in experiments are of analytical grade and available from commercial sources. Tetraethylxysilane (98.6%), diethanol amine (99%), methyl acrylate (98.5%), p-toluenesulfonic acid (99%), FeSO_4_·7H_2_O, and FeCl_3_·6H_2_O were all purchased from Shanghai Macklin Biochemical Co., Ltd., Shanghai, China.

### 3.2. Preparation of Fe_3_O_4_@HAAP

The Fe^2+^ and Fe^3+^ mixed solution was obtained by dissolving 2.7 g FeSO_4_·7H_2_O and 5.7 g FeCl_3_·6H_2_O into 100 mL deionized water. Ammonia water was added drop by drop into the solution and adjusted to a pH of 10. Then, the reaction lasted 30 min at 80 °C. The product (Fe_3_O_4_) was drawn out by a magnet and washed several times with ethanol and distilled water.

The prepared Fe_3_O_4_ was evenly dispersed in the mixture of ethanol and deionized water (volume ratio: 4:1). Ammonia water (28%) and tetraethylxysilane (98.6%) were added to the solution in turn and reacted for 6 h. Finally, Fe_3_O_4_@SiO_2_ was harvested by magnetic separation and freeze-drying.

Methyl N (10 mL) and n-dihydroxyethyl-3-aminopropionate (9.1 mL) were produced via mixing methyl alcohol, diethanolamine, and methyl acrylate adequately in a three-mouth flask with a reaction for 4 h at 35 °C, then heated up to 85 °C for 80 min. Trimethylolpropane (4.43 g) and p-toluenesulfonic acid (0.1 g) reacted with methyl N, n-dihydroxyethyl-3-aminopropionate to form hyperbranched amino acid polymer (HAAP) at specific conditions (120 °C for 3 h and then 100 °C for 1 h). With the addition of maleic anhydride (3.56 g) and p-toluenesulfonic acid (0.16 g), the target product Fe_3_O_4_@HAAP was obtained via the assembly of HAAP and Fe_3_O_4_@SiO_2_.

### 3.3. Characterization of Fe_3_O_4_@HAAP

The infrared spectrogram analysis of samples (Fe_3_O_4_, HAAP, and Fe_3_O_4_@HAAP) was carried out with a Fourier transform infrared spectrometer (FTIR, IRTracer-100, Shimadzu, Kyoto, Japan). The resolution was 1 cm^−1^, and the wavelength range was 400~4500 cm^−1^. X-ray photoelectron spectroscopy of Fe_3_O_4_@HAAP was tested via an Escalab 250 Xi spectrometer (XPS, Thermo Fisher Scientific Inc., Waltham, MA, USA) with a monochromated source of X-rays (Al Kα, 1486.6 photo energy) as the excitation source. The surface morphology and element composition were observed with a scanning electron microscope (SEM) equipped with energy dispersive X-ray spectroscopy (EDS, FlexSEM 1000, Hitachi, Tokyo, Japan) with an operating voltage of 3 kV. The magnetic property of Fe_3_O_4_@HAAP was examined using a vibrating-sample magnetometer (VSM, LakeShore 7404, Columbus, OH, USA) and the test magnetic moment range is 5 × 10^−7^ to 10^3^ emu with the maximum magnetic field being 2.17 T. The zeta potential of Fe_3_O_4_@HAAP was detected by a zeta potential analyzer (2000HSA, Malvern, UK).

### 3.4. Application in Wastewater Treatment

In order to verify the flocculation property of Fe_3_O_4_@HAAP, kaolin suspension simulated wastewater was used as the treatment target. Different reaction conditions, such as Fe_3_O_4_@HAAP dosage, pH, and kaolin concentration, were explored. The Fe_3_O_4_@HAAP dosage range was 0~120 mg/L. The pH range was 4~10. The kaolin concentration range was 0.5~3 g/L. The reaction time was 0~30 min. In order to investigate the influence of reaction time and then discuss the flocculation kinetics, the reaction lasted for 9 h with timed sampling. In order to test the application effect in the actual water, Fe_3_O_4_@HAAP and Fe_3_O_4_ were added to the target water, respectively. The reaction was conducted under the conditions that the Fe_3_O_4_@HAAP dosage was 50 mg/L, pH = 5, and the reaction time was 30 min. Since the turbidity in the natural water was much lower than the experimental concentration, kaolin was added to the original water to bring the turbidity up to 1300~1450 NTU. In order to investigate the stability performance of Fe_3_O_4_@HAAP, the material was recycled and reused five times in kaolin treatment.

### 3.5. Interaction Energy Analysis of Fe_3_O_4_@HAAP and Kaolin

The Derjaguin–Landau–Verwey–Overbeek (DLVO) and extended DLVO (EDLVO) theories were used to analyze the mechanism of magnetic flocculation between Fe_3_O_4_@HAAP and kaolin. The interaction energies between magnetic aggregates include van der Waals (U*_vdw_*), electrostatic interaction (U*_EI_*), and Lewis acid–base interaction (U*_AB_*) [7]. Herein, in the DLVO theory, the total interaction energy (U*_DLVO_*) between the surfaces of interacting particles was simulated by the sum of U*_vdw_* and U*_EI_*. In the EDLVO theory, the total interaction energy (U*_EDLVO_*) was simulated by the sum of U*_DLVO_* and U*_AB_*. The relevant calculation formulae for computing the total interaction energies are as follows [36,53,54]:

(1) $$U_{vdw}=-\frac{A_{132}R_{1}R_{2}}{6D(R_{1}{+R}_{2})}$$

(2) $$U_{EI}=\frac{\pi \varepsilon R_{1}R_{2}(\varphi_{1}^{2}+\varphi_{2}^{2})}{\left(R_{1}{+R}_{2}\right)}\left[\frac{{2\varphi }_{1}\varphi_{2}}{\varphi_{1}^{2}+{\psi \varphi }_{2}^{2}}\ln\frac{1+exp(-\kappa D)}{1-exp(-\kappa D)}+\ln\left\{1-exp(-2\kappa D)\right\}\right]$$

(3) $$U_{AB}=4\pi \frac{R_{1}R_{2}}{R_{1}{+R}_{2}}\lambda_{AB}\Delta G_{h_{0}}^{AB}exp\left[\left(h_{0}-D\right)/\lambda_{AB}\right]$$

where all the relevant parameters for equations are summarized in Table 2.

### 3.6. Analysis Methods

The turbidity meter (WGZ-800, Shanghai Xinrui Instrument Co., Ltd., Shanghai, China) was used to measure turbidity during the experiments and the removing efficiency was calculated with Equation (4):

(4) $$Removing\;efficiency\;=\;\frac{Initial\;turbidity\;-Sample\;turbidity}{Initial\;turbidity}\times 100\%$$

After the experiments, the supernatant was removed from the suspensions. The magnetic particle–kaolin aggregates were collected and dispersed in deionized water (10 mL). The kaolin particles were detached from the Fe_3_O_4_ particles by employing an ultrasonic generator (50 Hz, 1200 W) for 5 min. The Fe_3_O_4_ particles were collected with a permanent (50 mm L × 50 mm W × 25 mm H, 0.38 T) and then washed three times with 10 mL deionized water. The collected Fe_3_O_4_ particles were vacuum-dried to obtain recycled Fe_3_O_4_ powder. The recycling efficiency of Fe_3_O_4_ or Fe_3_O_4_@HAAP was calculated using Equation (5):

(5) $$Recycling\;efficiency=\frac{m_{i}}{m_{o}}\times 100\%$$

where *m_o_* (g) and *m_i_* (g) are the initial quality of Fe_3_O_4_ or Fe_3_O_4_@HAAP, and the quality after recovery time *i* (*i* = 1, 2, 3… 5), respectively. The recovered Fe_3_O_4_ or Fe_3_O_4_@HAAP was employed in the fresh kaolin suspensions immediately without any pre-treatment.

### 3.7. Statistical Analysis

The significant differences in the experiment data were determined by one-way analysis of variance (ANOVA) via IBM SPSS 20 (SPSS Inc., Chicago, IL, USA). A value of *p* < 0.05 was considered to be significantly different.

## 4. Conclusions

A novel three-dimensional magnetic composite of Fe_3_O_4_@HAAP was assembled by hyperbranched amino acid composites and Fe_3_O_4_. Through characterization, it was found that the hyperbranched polymer successfully linked with Fe_3_O_4_ and exhibited a lotus leaf-like morphological structure at the micro level. The superparamagnetic properties still existed and were easily affected by magnetic fields. Fe_3_O_4_@HAAP exhibited excellent turbidity (kaolin) removing efficiency and the removing efficiency reached 94.83% when the Fe_3_O_4_@HAAP dosage was 50 mg/L, pH = 5, and the kaolin concentration was 1 g/L. The flocculation of kaolin with Fe_3_O_4_@HAAP was a rapid process; the removing efficiency was 83.85% and 91.96% at 0.25 h, as the dosages were 10 and 50 mg/L. Fe_3_O_4_@HAAP proved to have such high stability that the recycling and removing efficiency were over 97% and 92.6%, respectively, after five cycles. In DLVO and EDLVO theory analysis, the mutual potential energy of kaolin–kaolin particles were dominated by U*_EI_*, which showed electrostatic repulsion at short distances (<30 nm) and electrostatic attraction at long distances (>30 nm). When Fe_3_O_4_@HAAP was added to kaolin solution, an external magnetic field would cause the energy barrier of kaolin particles to be crossed, and the kaolin particles were destabilized and aggregated. The above results indicated that Fe_3_O_4_@HAAP would be more competitive in the treatment of ecological water due to its biodegradability and environmental friendliness in the future.

## Figures and Tables

**Table 1:** EDS values of Fe_3_O_4_@HAAP.

C		N		O		Si		Fe	
Wt%	At%	Wt%	At%	Wt%	At%	Wt%	At%	Wt%	At%
52.78	64.33	6.90	7.21	26.10	23.88	3.26	1.70	10.96	2.874
38.58	53.81	4.58	5.48	29.76	31.16	4.83	2.88	22.25	6.68

**Table 2:** The parameters for DLVO and EDLVO equations [7,36].

Symbols	Parameters
R	The radius of magnetic materials and kaolin
A_132_	The effective Hamaker constant (J) for particles (1) interacting with particles (2) in the aqueous medium (3)
h_0_	The minimum equilibrium distance due to the Born repulsion, 0.157 nm
D	The separation distance between the two interacting particles (nm)
λ	The correlation length of molecules in a liquid medium, 0.6 nm
κ	The inverse Debye length (m^−1^), 0.11 nm^−1^
N_A_	Avogadro’s number, 6.02 × 10^23^ mol^−1^
exp	Unit charge, 1.602 × 10^−19^ C
ε	the dielectric constant of the solution, (80 × 8.854 × 10^−12^ C^2^/J/m for aqueous)
K	Boltzmann constant, 1.38 × 10^−23^ J·K^−1^
T	The absolute temperature taken as 298 K
φ	The magnetic materials and kaolin surface potentials (V), depending on the zeta potential

## Data Availability

The data presented in this study are available on request from the corresponding author.
